# Early detection of cold stress to prevent hypothermia: A narrative review

**DOI:** 10.1177/20503121231172866

**Published:** 2023-05-13

**Authors:** Michiko Kyokan, Nathalie Bochaton, Veena Jirapaet, Riccardo E Pfister

**Affiliations:** 1 Institute of Global Health, University of Geneva, Geneve, Switzerland; 2 Geneva University Hospitals and Geneva University, Geneve, Switzerland; 3 Faculty of Nursing, Chulalongkorn University, Bangkok, Thailand

**Keywords:** Neonate, thermal care, cold stress, hypothermia, temperature monitoring, low-resource settings

## Abstract

Temperature monitoring is essential for assessing neonates and providing appropriate neonatal thermal care. Thermoneutrality is defined as the environmental temperature range within which the oxygen and metabolic consumptions are minimum to maintain normal body temperature. When neonates are in an environment below thermoneutral temperature, they respond by vasoconstriction to minimise heat losses, followed by a rise in metabolic rate to increase heat production. This condition, physiologically termed cold stress, usually occurs before hypothermia. In addition to standard axillary or rectal temperature monitoring by a thermometer, cold stress can be detected by monitoring peripheral hand or foot temperature, even by hand-touch. However, this simple method remains undervalued and generally recommended only as a second and lesser choice in clinical practice. This review presents the concepts of thermoneutrality and cold stress and highlights the importance of early detection of cold stress before hypothermia occurs. The authors suggest systematic clinical determination of hand and foot temperatures by hand-touch for early detection of physiological cold stress, in addition to monitoring core temperature for detection of established hypothermia, particularly in low-resource settings.

## Introduction

In 2020, 2.4 million neonates died globally, three-quarters of them in the first day of life.^1^ Neonatal mortality accounts for 47% of all child deaths under the age of 5 years globally, up from 40% in 1990 with slower reduction pace than under-five mortality. Hypothermia (<36.5°C) has been widely regarded as a major contributory factor in neonatal mortality and morbidity in low-resource settings^2–4^ where high prevalence of neonatal hypothermia has been reported.^5,6^ Preterm infants (babies born before 37 weeks of gestation^7^) and low-birth-weight (weight < 2500 g at birth^7^) are prone to hypothermia due to their physiologic immaturity and physical disadvantages.^6,8–12^ A recent study from Ethiopia reported that 80% of preterm infants who died had hypothermia.^13^ Hypothermia is preventable in the majority of neonates because it is not a complication of prematurity itself but more the result of inadequate thermal care.^14,15^

A warm ambient environment (>28°C) increases the chances of survival among preterm and low-birth-weight neonates.^16^ The concept of a thermoneutral zone was introduced in neonatal care more than half a century ago.^17^ It is generally defined as the environmental temperature range within which the oxygen and substrate consumption for thermoregulation of homeotherms is minimal.^18,19^ In an environment below such temperature, neonates respond with vasoconstriction to minimise heat losses, followed by a rise in metabolic rate to increase heat production.^20^ In physiological terms, this condition is defined as cold stress, and it triggers additional metabolic defence mechanisms using more oxygen and glucose,^21^ leading to hypothermia only when these are finally exhausted. Therefore, early detection of cold stress is essential for timely intervention to prevent hypothermia.

Cold stress can be detected by monitoring peripheral temperature, typically hand or foot temperature, even by hand-touch. It is because cold stress first induces vasoconstriction to reduce heat loss and hands and feet receive less perfusion and become colder. The importance of monitoring hand and foot temperatures by hand-touch for early detection of cold stress has been reported over decades.^22,23^ However, this simple method remains undervalued and generally recommended only as a second and lesser choice in clinical practice.^16,24^ In current clinical practice, axillary or rectal temperature is commonly measured to confirm normothermia (36.5°C–37.5°C), or detect hyperthermia (>37.5°C) or hypothermia.^4,25,26^ This measurement of a single temperature detects established body temperature only and gives no clue about the dynamic physiological efforts to maintain it. Hence, it is important to monitor peripheral hand or foot temperature by hand-touch in addition to standard axillary or rectal temperature monitoring by a thermometer. The purpose of this review is to (1) present the concepts of thermoneutrality and cold stress, (2) highlight the importance of early detection of cold stress before hypothermia occurs and (3) suggest a practical method to detect cold stress for optimal thermal care in neonates, particularly in low-resource settings. In this review, our focus is on early detection of cold stress by simple methods to prevent hypothermia among newborn infants.

## Methodology

We performed a literature search in the following online electronic databases: PubMed, Embase and Google Scholar, using the keywords and logical combinations: ‘Infant, Newborn’[MeSH Terms], ‘hypothermia’[MeSH Terms], ‘skin temperature’[MeSH Terms], ‘hypothermia’, ‘cold stress’ and ‘thermoneutrality’. Articles were retrieved and selected based on relevance to the research objectives. Articles in English or French were included. We have opted not to restrict the search to low-resource settings and a specific time frame to maximise the number of entries. Systematic reviews, randomised controlled trials and quasi-randomised controlled trials were included. Descriptive observational studies, non-systematic reviews, qualitative studies, cohort studies, case studies and other lower quality designs were included when relevant. Included articles’ reference lists were screened for additional articles of interest.

## Thermoneutrality and cold stress

**Figure 1.**
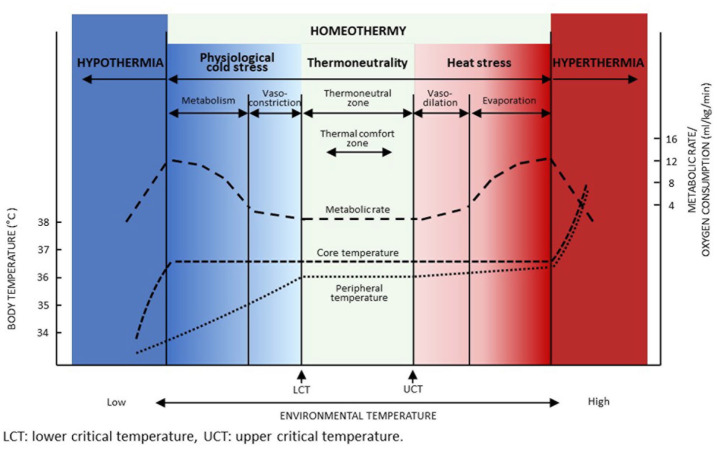
Schematic representation of the thermoneutral zone in a homeotherm human. Source: Freely adapted from Adams^29^; Brück^30^; Gordon^19^.

The ambient temperature plays a key role in thermal care. As shown in Figure 1, the ambient lower critical temperature indicates the limit below which core body temperature must be maintained through additional metabolic heat production. Similarly, on the opposite side of the scale, the ambient upper critical temperature indicates the limit above which maintenance of core body temperature requires increased temperature losses through evaporation.^18,19^ The range between lower and upper critical temperatures is called the thermoneutral zone. Within this zone, a stable core body temperature is maintained by thermoregulatory efforts at a resting metabolic rate, with minimal oxygen and substrate consumption. In neonates, especially preterm infants, the lower critical temperature is considerably higher and the thermoneutral zone is much narrower than that of a child or adult due to their unfavourable surface to core ratio, immature skin barrier and reduced subcutaneous fat insulation.^27^ The lower the body weight, gestational age and postnatal age, the narrower the thermoneutral zone.^28^

In physiological terms, cold stress is defined as a condition in which the environmental temperature is below the lower critical temperature for thermoneutrality (18; see Figure 1). However, cold stress has been used with a variety of meanings in neonatal thermal care. In the World Health Organization (WHO) neonatal thermal care guidelines, cold stress is used synonymously with mild hypothermia, an axillary or rectal temperature of 36.0°C–36.4°C.^16^ Hence, this definition has been used in several publications citing the WHO’s guidelines.^31–33^ Although some researchers did not provide the specific definition of cold stress in their papers, from the context, it seems that cold stress was not used synonymously with mild hypothermia but as a condition being exposed to cold environmental temperature.^34,35^ Finally others, explained cold stress as the physiological response to cold, which implies they used cold stress in physiological terms.^23,36,37^ In this review, we use physiological cold stress, defined as a condition in which the environmental temperature is below the lower critical temperature for thermoneutrality.

Physiological cold stress first induces vasoconstriction, to reduce heat loss, and then leads to increased metabolic heat production. Under physiological cold stress, the skin of the hands and feet are less perfused and become colder because of vasoconstriction. Arteriovenous anastomoses, which play a major role in thermoregulation, are abundant in the glabrous skin of the hands and feet.^38^ They consist in direct connections between small arteries and small veins without capillary section^39^; their vasoconstriction and vasodilatation is used to regulate body temperature.

When reduction of heat loss by vasoconstriction is insufficient to regulate body temperature, metabolic heat production will be ‘turned on’. Metabolic heat production consumes excess oxygen and energy, competing with the need to maintain essential vital functions and growth, and if insufficient, will eventually lead to hypothermia.^40^ Increased metabolic rate burdens major yet vulnerable physiological perinatal adaptation processes, causing or worsening illnesses such as respiratory distress and leading to hypoglycaemia and energy failure.^41^

## Core and skin temperatures

Body temperatures can be measured as core or skin temperatures.^42^ Strictly speaking, core temperature is defined as the temperature in the hypothalamus, where the natural thermostat is situated, and, by association, in the vital internal organs.^43^ As we cannot measure the temperature inside these organs, the pulmonary artery, distal oesophagus, bladder and nasopharynx are used as the closest proxy for core body temperature.^44^ However, even these measurement sites require invasive techniques that are unrealistic in most clinical settings. Therefore, rectal and axillary temperatures are commonly used.^45^

**Figure 2.**
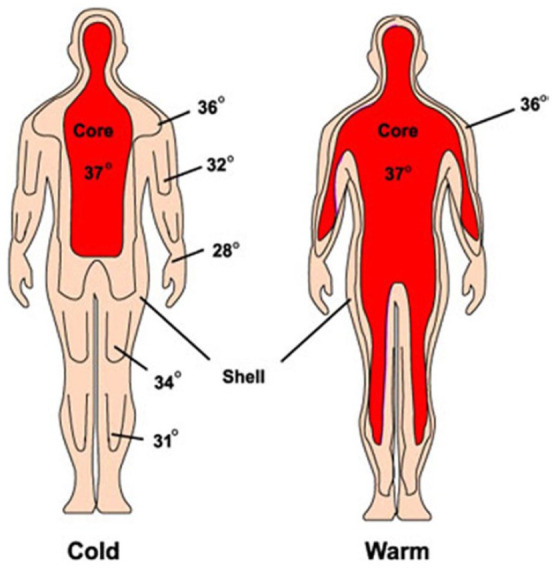
Core and skin surface temperature distribution. Source: Reprinted from Tamura et al.^42^ Copyright 2018 by the Japanese Society for Medical and Biological Engineering.

Skin temperature is generally lower than core body temperature and is influenced by natural body insulation, physiological regulatory mechanisms such as skin blood flow, sweating and evaporation from immature or wet skin. External factors such as airflow, thermal radiation and insulation by clothing also influence skin temperature.^40^ As a physical rule, body heat dissipates from warmer to colder areas, and depends on heat conductance and distance. In general for the body, the heat of the core is therefore dissipated into the environment through the skin and mucosal surfaces, and gradients appear as shown in Figure 2.^42^ Skin temperature is heterogeneous and varies by location; it is higher over arteries, such as the axillary artery, where its proximity to the heart keeps the temperature closer to core temperature. When the skin’s surface is enclosed by cloth, other insulation or a skin fold, heat dissipation is reduced and skin temperature comes closer to core temperature. This occurs, for example, when measuring temperature in the axilla covered by the adducted arm.^46^ A very useful feature of skin surface temperature, however, is its variability, which largely reflects physiological regulatory mechanisms.

## Hand and foot temperatures

For optimal thermal care, it is essential to determine whether the neonate is in its thermoneutral zone and therefore at resting metabolic rate. Physiological cold stress and even more so established hypothermia need to be avoided. Although oxygen consumption measurements enable estimation of the metabolic rate and can confirm that a neonate is in its thermoneutral zone,^47^ it is not a practical option especially in low-resource settings.

The temperature difference between the core and periphery can reveal vasomotor thermal responses.^48^ During physiological cold stress, peripheral temperatures fall due to vasoconstriction, particularly in hands and feet, well before core temperature falls and hypothermia establishes.^37,45,49^ Hence, monitoring hand and foot temperatures may be a simple and good indicator of physiological cold stress^50–52^ whereas core temperature detects established hypothermia or hyperthermia.

## A practical monitoring method to detect cold stress

An Indian paediatrician once said ‘[The] sole is the mirror of [the] newborn’s health’.^22^ Despite this, it remains a common belief that cold feet in neonates are an ordinary condition and just need local warming or protection such as socks. On the contrary, they should be considered a critical sign of cold stress. For the neonate, this is an uncomfortable, heat-sparing condition that occurs below the lower critical temperature with wasted oxygen and energy.^49^

In some high-resource settings, the foot temperature of neonates in incubators or under radiant warmers is continuously monitored, in addition to core temperature, using a second thermistor probe, in order to detect physiological cold stress early.^49,53^ However, such practices are not widespread because of technical complexity, limited resources and the need for knowledgeable interpretation. It may not be necessary to measure the temperature of the extremities continuously and with high precision to detect cold stress. A simple, frequent check of hand and foot temperatures by hand-touch allows early detection.

Singh et al.^54^ reported the precision of the hand-touch as evaluated by three paediatricians in 50 full-term healthy neonates in India. At three sites (abdomen, foot, forehead), temperatures <36.5°C were accurately detected by hand-touch compared against electronic thermometer. Another study conducted by Ellis et al.^55^ assessed the validity of hand-touch by health workers for the detection of cold stress (defined as 36°C–37°C in their study) and hypothermia (defined as <36°C in their study) in 250 neonates in Nepal. They compared the findings of each observer using palpation with axillary mercury thermometer. They categorised a warm abdomen and feet to indicate normothermia, a warm abdomen with cold feet to indicate cold stress, and cold abdomen and cold feet to indicate hypothermia. The specificity of palpation to detect hypothermia was 93%–100%, but the sensitivity was 11%–42%. Sensitivity improves considerably when only feet assessment was used.

Furthermore, Agarwal et al.^56^ examined the diagnostic accuracy of hand-touch to detect hypothermia against axillary digital thermometer by a trained, non-medical field supervisor of community health volunteers among 148 neonates in India. Hypothermia assessed by hand-touch method showed a high diagnostic accuracy when compared against axillary digital thermometer (kappa 0.65–0.81; sensitivity 74%; specificity 96.7%). Later, Agarwal et al.^56^ assessed again the validity of hand-touch to measure hypothermia compared against axillary digital thermometry by supervisors of slum-based health volunteers in 152 neonates in India. The authors found that hand-touch method had moderate diagnostic accuracy when compared with axillary digital thermometry (kappa: 0.38, sensitivity: 74.5%, specificity: 68.5%).

Finally, Tuitui et al.^57^ conducted a study to examine the diagnostic validity of hand-touch method against axillary mercury thermometer and tympanic thermometer for detecting neonatal hypothermia among 100 full-term neonates, delivered within 24 h in a hospital in Nepal. The sensitivity and specificity of the hand-touch method for detection of neonatal hypothermia were 95.6% and 70.1% against axillary mercury thermometer and 76.6% and 83% against the tympanic thermometer, respectively.

## Limitations

Like other narrative reviews, this review did not follow a specific set of evidence-based criteria to select or evaluate the included references, potentially introducing bias and preventing replicability. This review was also limited by the studies performed more than 5 years ago, especially for the validation of the diagnostic accuracy of the hand-touch method to detect cold stress and hypothermia and reflects the fact that only few studies have been conducted in the neonates in low-resource settings. Nevertheless, we believe there is enough evidence to suggest hand-touch to monitor hand and foot temperatures for detection of cold stress and hypothermia in both hospital and community settings. Although most studies examined the accuracy of the hand-touch method to detect hypothermia (low core temperature), the method has potential for early detection of physiological cold stress (low peripheral temperature) to prevent hypothermia. We therefore suggest systematic and regular monitoring of hand and foot temperatures for early detection of physiological cold stress, in addition to monitoring core temperature for detection of established hypothermia. Repeated hand-touch may further increase an already considerable sensitivity for such a cheap method.

## Conclusions

In the WHO neonatal thermal care guidelines, cold stress is defined as axillary or rectal temperature of 36.0°C–36.4°C, and is used synonymously with the term ‘mild hypothermia’. We believe this downplayed definition of cold stress and mild hypothermia leads healthcare professionals, particularly those unknowledgeable of neonatal physiology, to underestimate the seriousness of the condition. Indeed, any hypothermia, even if mild, results from the exhaustion of metabolic compensatory processes. It thus appears obvious that only a clear distinction between cold stress and hypothermia allows early intervention before exhaustion of metabolic thermogenesis. Metabolic efforts to maintain core temperature waste vital glucose and oxygen and must be considered pathogenic as established central hypothermia. Clearly, monitoring an axillary or rectal temperature alone is insufficient to prevent hypothermia. The strong dose-response association between hypothermia and neonatal death is epidemiological confirmation of the importance of early prevention.

The WHO recommends checking skin temperature by hand-touch only at home if there is no thermometer available. This contextual recommendation may falsely suggest that hand-touch temperature assessment is of lower ‘value’ than a technical assessment with a thermometer. We believe the 1997 WHO guidelines, which are still cited frequently today, urgently need revision to ensure they distinguish clearly between physiological cold stress and hypothermia. By doing so, healthcare professionals may understand physiological cold stress as a serious neonatal condition leading to death, and not as a ‘mild’ condition likely to recover spontaneously. To prevent physiological cold stress, hypothermia and hypothermia-associated morbidity and mortality, it is essential that healthcare professionals are educated in the thermal physiology of neonates.

The hand-touch temperature assessment is an efficient, simple and economical method to detect physiological cold stress. This assessment complements existing methods of measuring body temperature and could be implemented not only in low-resource settings but in most healthcare settings such as during skin to skin care. Further scientific evaluation appears to be necessary to assess how accurately repeated routine hand-touch can detect physiological cold stress and the learning curve required to achieve accurate detection, but also whether it indeed reduces neonatal mortality in clinical practice in low-resource settings.
